# P-1674. Comparative study of advance practice providers and physician prescribing practices for complicated intra-abdominal infections in patients hospitalized at a safety-net community hospital

**DOI:** 10.1093/ofid/ofae631.1840

**Published:** 2025-01-29

**Authors:** Aakash Balaji, Ben Pomerantz, Jessica Hua, Dylan Huber, Mirza Ali, Lawrence Sanchez, Alfredo J Mena Lora

**Affiliations:** University of Illinois, Chicago, Illinois; University of Illinois at Chicago, Chicago, Illinois; University of Illinois at Chicago, Chicago, Illinois; Saint Anthony Hospital, Chicago, Illinois; Saint Anthony Hospital, Chicago, Illinois; Saint Anthony Hospital, Chicago, Illinois; University of Illinois Chicago, Chicago, Illinois

## Abstract

**Background:**

The expansion of advanced practice providers (APPs) in the inpatient setting warrants understanding of their antimicrobial prescribing practices in these settings to help adapt antimicrobial stewardship (ASP) strategies. Variability in APP training programs further complicates the landscape. This study aims to investigate disparities in antibiotic prescription practices for complicated intra-abdominal infections (cIAI) between APPs and physicians.

Figure 1

**Figure:**
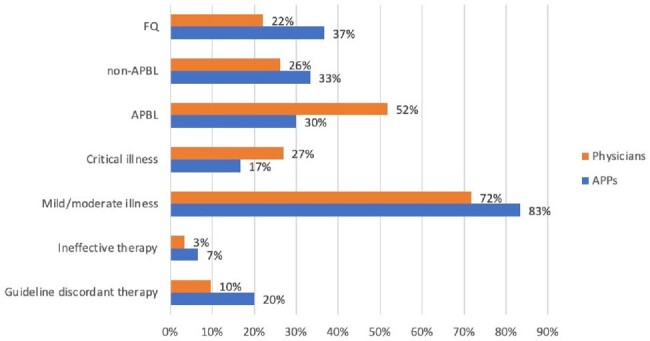

clAl severity and empiric antimicrobial selection by physicians and APPs

**Methods:**

A retrospective review of antibiotic orders was conducted at a 151-bed community hospital. ASP prospective audit and feedback (PAF) data from July 2022 to June 2023 was reviewed, including initial antibiotic selection, duration, and guideline concordance for APPs and physicians. Guideline adherence, antimicrobial effectiveness, and days of therapy (DOT) per 1000 patient days was compared between APPs and physicians.

FIgure 2

**Figure:**
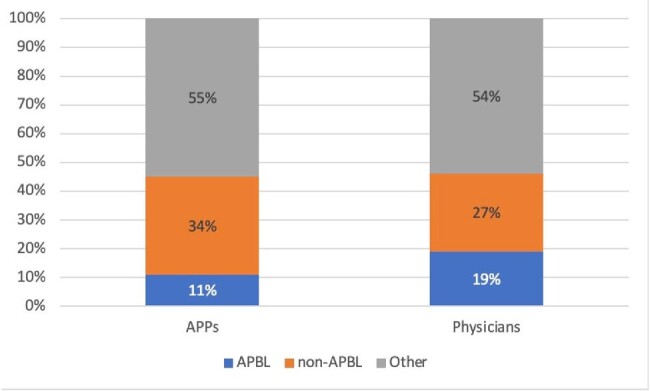

Empiric antimicrobial selection by physicians and APPs

**Results:**

A total of 175 initial empiric PAF entries for cIAI were reviewed, of which 145 were from physicians and 30 from APPs. Guideline non-adherence was 10% (14) for physicians and 20% (6) for APPs. Additionally, 3% (5) of physician and 7% (2) of APP prescriptions were ineffective. Critical illness was higher for physician cases (Figure 1). Physicians prescribed more antipseudomonal beta-lactam (APBLs) than APPs (52% vs 30%). APPs prescribed more quinolones (FQ) than physicians (33% vs 26%). Of the patients treated with FQs by APPs, 37% had allergies to beta-lactams, compared to 22% of patients treated by physicians. Total DOT/1000 during the study period was 145. DOT/1000 was 44 for physicians, of which 11% were anti-pseudomonal beta lactams (APBLs) and 34% non-APBLs. DOT/1000 was 21 for APPs, of which 19% were anti-pseudomonal beta lactams (APBLs) and 27% non-APBLs (Figure 2).

**Conclusion:**

Empiric antimicrobial selection for cIAI differed between APPs and physicians, with APP showing higher quinolone use, a non-preferred agent, and almost double the level of non-adherence to guidelines. These findings underscore the need for tailored ASP education and interventions that address the unique practices and educational backgrounds of APPs, aiming to standardizing and optimizing antimicrobial use. APPs may require specific education for cIAI and ASP guidance.

**Disclosures:**

**All Authors**: No reported disclosures

